# Experiential Avoidance in Advanced Cancer: a Mixed-Methods Systematic Review

**DOI:** 10.1007/s12529-022-10131-4

**Published:** 2022-10-25

**Authors:** Sarah Davis, Marc Serfaty, Joe Low, Megan Armstrong, Nuriye Kupeli, Anne Lanceley

**Affiliations:** 1 Division of Psychiatry, Marie Curie Palliative Care Research Dept, University College London, Maple House, Tottenham Court Road, London, W1T 7NF UK; 2 Division of Psychiatry, University College London, London, UK; 3 Primary Care and Population Health, University College London, London, UK; 4 EGA Institute for Women’s Health, Department of Women’s Cancer, University College London, London, UK

**Keywords:** Advanced cancer, Experiential avoidance, Mixed methods, Psycho-oncology, Systematic review

## Abstract

**Background:**

People with advanced cancer experience psychological distress due to physical symptoms, functional decline, and a limited prognosis. Difficult thoughts, feelings, and emotions may exacerbate distress and lead to avoidance of these experiences which is sometimes referred to as experiential avoidance (EA). Advanced cancer patients may be more likely to engage in EA especially when no obvious solutions to their problems exist. This study aims to examine the terms used to describe EA, the processes that might indicate EA, associations between EA and psychological distress, and to understand why individuals might engage in EA.

**Methods:**

A mixed-methods review. Literature search of Medline, Embase, Psych INFO, and CINAHL 1980–October 2019. Inclusion: adults ≥ 18 years; advanced cancer not amenable to cure. Exclusion: no measures of EA or psychological distress. Risk of bias and study quality assessed. Evidence of statistical techniques collected. Themes coded, grouped, and developed based on meaning.

**Results:**

Nineteen studies identified, 13 quantitative studies and 6 qualitative. The quantitative of which 6 compared early-stage cancers with advanced cancers and examined subscales of EA alongside mood, quality of life, and psychological distress. EA covers a range or terms of which ‘avoidant coping’ is the commonest. EA is manifest as cognitive, behavioural, and emotional avoidance. A thematic synthesis suggests the function of EA is to protect people from distress, and from confronting or expressing difficult emotions by avoiding communication about cancer, controlling negative information, and maintaining normality and hope and optimism.

**Conclusions:**

EA may be beneficial in the short term to alleviate distress, but in the longer term, it can impair function and limit engagement in life. Greater clinical awareness of the complexity of EA behaviours is needed. Clinicians and researchers should define EA precisely and be aware of the function it may serve in the short and longer term. Future research studies may consider using specific measures of EA as a primary outcome, to assess the impact of psychological interventions such as ACT.

**Supplementary Information:**

The online version contains supplementary material available at 10.1007/s12529-022-10131-4.

## Introduction

Improvements in medical treatments mean that people are living longer with advanced, non-curative cancers [1]. An uncertain prognosis, limited life expectancy, and an increasing symptom burden can make life difficult in advanced cancer and lead to psychological distress [2–4]. Distress is often multifactorial, an unpleasant emotional experience that is psychological, social, and/or spiritual in nature, which can interfere with the ability to cope [5]. Negative thoughts may exacerbate the problem and lead to avoidance of social and psychological issues [6]. Individuals with advanced cancer are challenged to engage fully in life whilst living with symptomatic disease and closeness to death [7]. Individuals challenged by a stressor beyond their resources may use cognitive, behavioural, and emotional strategies to manage internal and external demands [1]. Behaviours directed at avoiding the problem to prevent experiencing distress or to lessen emotional reactions are emotion focused and known as ‘avoidance coping’ [8]. Avoidance is identified as an important psychological response [9], and a link is made between avoidance and anxiety [10].

The link between avoidance and anxiety was first alluded to by Freud [11] in psychodynamic therapy, as he noticed that people were sometimes unable to remain in contact with upsetting material, and Freud suggested that as a result, people used repression as a way of coping with this distress [11]. Cognitive and dialectical therapy identified avoidance of unpleasant experiences and contexts in which they arose as a problematic way of dealing with distress [12]. Evidence exists in the literature of the association between avoidance and anxiety and maladaptive psychological functioning [12–18].

A wider definition of avoidance behaviours has developed in the psychological literature called experiential avoidance (EA) which consists of two related parts: (a) the unwillingness to remain in contact with troublesome experiences (including bodily sensations, emotions, thoughts, memories, and behavioural predispositions) and (b) action taken to alter these experiences or the events that elicit them which includes all forms of avoidance and escape [19].

EA encompasses different cognitive, behavioural, and emotional avoidance behaviours which over time have been identified, measured, and labelled in both the coping and psycho-oncology literature [20, 21]. The subscales of avoidance that are measured include escape, denial, behavioural and mental disengagement, and wishful thinking [22].

**Fig. 1:**
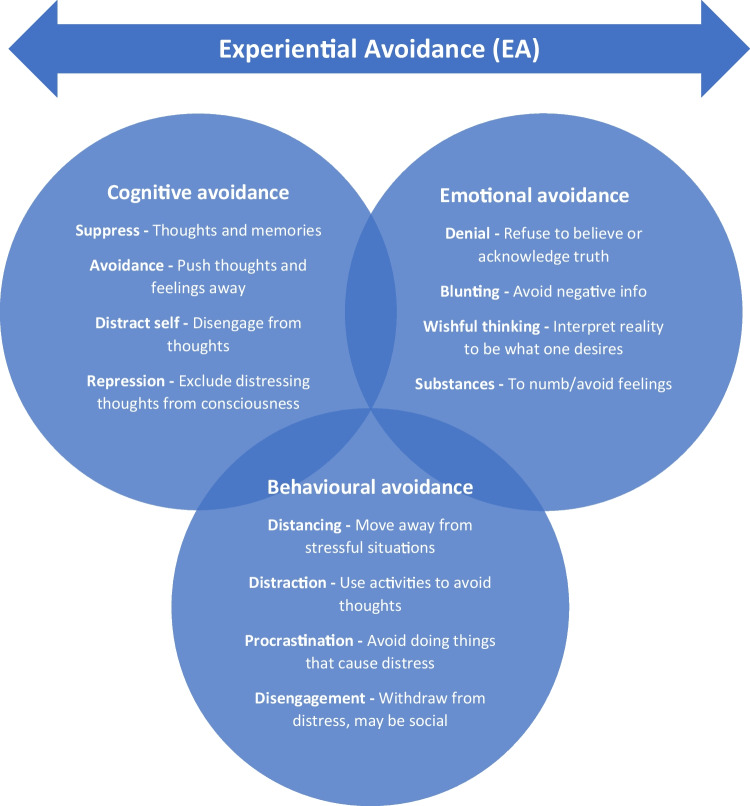
Types of avoidance in EA

For the purposes of this review and for ease of use regarding definitions, we have categorised and defined avoidance in the following ways. *Cognitive avoidance* refers to attempts to suppress, avoid, disengage, and distract from thoughts and memories that may be intrusive and cause distress and worry [9, 23–25]. *Behavioural avoidance* refers to actions to physically distance, disengage, distract, and prevent contact with unwelcome experiences [26, 27]. *Emotional avoidance* refers to actions to alleviate or manage the distress difficult experiences may cause and includes denial, repression, wishful thinking, blunting—only attending to positive information—and using substances like alcohol or activities such as sleep to avoid or numb experiences [23, 24, 26, 27]. The different types of avoidance that make up EA are depicted in Fig. 1.

EA is considered to be a core psychopathological process in various empirically based modern cognitive behavioural therapies, for example, in Acceptance Commitment Therapy (ACT) [28] where it is acknowledged that attempts at avoidance may help to reduce and alleviate distress in the short term, but paradoxically, if it is used consistently over the longer term, it may reinforce the strength and frequency of upsetting experiences and concomitant distress. This is because avoidance strategies are often under verbal control and so are likely to influence further cognitions [18, 29]. EA also consists of taking action to avoid and escape from situations in which unpleasant experiences are evoked, thus restricting peoples’ activities [14]. EA becomes particularly problematic when it becomes habitual and energy is expended to prevent exposure to unwanted experiences [14]. In this circumstance, the avoidance is critical in the development and maintenance of psychopathology [19] and it is likely to lead to a lower quality of life [30].

A link has been established between cognitive and behavioural avoidance and higher levels of anxiety [31] and depressive symptoms [32].

A longitudinal cancer study found avoidance coping at baseline predicted chronic and acute stressors 4 years later and depressive symptoms 10 years later [21]. In mixed cancers at different stages, those who used cognitive and behavioural escape avoidance experienced more emotional distress [20] and denial in prostate, breast, and colon cancer was associated with higher anxiety and depression as well as cancer-related worries [33].

Our own research in advanced cancer found a negative association between acceptance and psychological morbidity which generated our interest to explore EA further [34]. Whilst there is evidence that avoidance exists and may be a maladaptive response to distress in cancer [35], there is no consensus on the definition and meaning of EA. This lack of definition makes it difficult to reach conclusions about the reasons for EA and its association with psychological distress in the cancer population.

The concept of EA is the focus and frame of our review in the advanced cancer population where recognition of the phenomenon and timely clinical intervention may be critically important. We propose to explore how EA is formulated in the advanced cancer literature: what terms are used and what measures or proxy measures are used to evaluate the phenomenon. Also, we are interested to see how those with advanced cancer articulate their experiences of illness insofar as these shed light on EA.

There has been no previous systematic review in this area, and our quantitative and qualitative review aims to [1] identify how EA is described in the advanced cancer literature and the processes that might indicate EA, [2] explore associations between EA and psychological distress, and [3] explore reasons for engaging in EA.

## Methods

The systematic review was registered on Prospero on 26^th^ July 2019 (registration number CRD42019139700; https://www.crd.york.ac.uk/PROSPERO/) and follows the Preferred Reporting Items for Systematic Reviews and Meta-Analyses [36].

We searched Embase, Medline, PsychINFO, CINAHL, conference abstracts, reference lists, and relevant reviews on the topic from January 1984 to October 2019. Our start date for the review was pegged to Lazarus and Folkman’s (1984) transactional theory of stress and coping [1], seminal work that established the link between coping and stress [37]. Search terms were generated using MeSH and subject headings to create the search term list for each database (Supplementary material 1). Terms were included for advanced cancer, experiential avoidance, and avoidance coping.

We included randomised controlled trials (RCTs) and non-randomised trials, observational studies, and qualitative and mixed-methods studies published in the English language. Studies in the advanced, recurrent, or metastatic adult [18 years and above] cancer population were included if participants were identified using recognised diagnostic criteria and/or participants were treated with palliative intent. Studies with a mix of early and late cancer stages III and IV were included if data were analysed separately. Studies were included if they used validated measures of EA or coping strategies—e.g. subscales of the COPE [27], or Brief COPE [38]—and measures of the effect on quality of life of psychological distress/mood disturbance.

One reviewer (SD) screened all titles and abstracts and selected relevant studies. SD completed a full text review of all the studies that met the inclusion criteria, and a second reviewer (MA) independently checked the included studies to ensure they met the criteria. Any disagreements between the two reviewers were resolved through discussion with the wider research team (AL/MS/JL). SD extracted data from included studies and was checked by MA.

### Outcomes of Interest

Our primary outcome is EA. As very few measures exist that measure EA directly, studies that used outcomes that assessed coping with stress were included. Outcome measures included the Coping Orientation to Problems Experienced (COPE/Brief COPE) [27, 38] and the Impact of Events Scale (IES) [24]. These measures contain subscales of types of avoidance that bear similarities with EA (Table 1). Our second outcome is psychological distress as it is commonly measured in association with coping styles in the cancer population [39].

**Table 1:** Outcome measures (subscales) of experiential avoidance (EA)

**Measure**	**EA**	**Example**
**COPE (60 items)** Costanzo 2006 Lutgendorf 2000 Sherman 2000	Mental disengagement Behavioural disengagement Denial Substances	Daydreaming, sleep I’ve been giving up attempt to cope, withdraw effort Don’t believe in situation, don’t acknowledge impact Use substances to numb feelings/feel better
**Brief COPE (28 items)** Nipp 2016 Sumpio 2017 Trevino 2012 Kershaw 2004	Self-distraction Behavioural disengagement Denial Venting Self-blame Alcohol/drug use	Use distraction to take mind off things Giving up, withdraw effort Don’t believe in situation, don’t acknowledge impact Expressing negative feelings Blame self for situation Use substances to numb feelings/feel better
**IES (15 items)** Sherman 2000 Costanzo 2006 Manne 2000	Avoidance cog + behavioural Intrusion—Intrusive thoughts	I stayed away from reminders of it I tried not to talk about it I had dreams about it Pictures popped into my mind

*COPE* Coping Orientations to Problems Experienced (Carver et al. [27]), *Brief COPE* shorter version of the COPE (Carver et al. [38]), *IES* Impact of Events Scale (Horowitz et al. [24])

We used a broad approach to assess psychological distress that included measures of mood disturbance, and psychiatric morbidity, as well as subscales of quality-of-life measures that assessed emotional well-being such as the FACT G [40].

### Quality Assessments

Two reviewers (SD and MA) independently assessed studies using the QualSyst tool [41], which was developed to standardise assessments of studies using different criteria to measure methodological quality and risk of bias at study and outcome level in both primary quantitative and qualitative research studies included in systematic reviews. The quantitative assessment consists of 14 items of which 11 items were applicable (no RCTs included). The qualitative assessment consists of 10 items of which all were applicable. Answers to questions in both assessments were categorised into one of three groups: fully, partially, or not answered. Study quality was assessed as good when the percentage of questions partially or fully answered combined exceeded 75%.

### Analysis

#### Quantitative Data

Data were extracted on statistical techniques used to explain the data including effect sizes, standard deviations, means, and *p* values. We planned, if appropriate, to do a meta-analysis where identified studies were similar in design and the tools that they used.

#### Qualitative Data

An analysis of quotations extracted from qualitative studies followed Thomas and Harden’s stepwise method for the synthesis of qualitative research results in systematic reviews [42]. Results were coded line by line according to meaning, organised into relevant areas and analytic themes developed by SD, which were reviewed and discussed with MA. A draft summary of the thematically organised grouped codes was discussed by other researchers (MS, JL, AL) who commented on the draft until a final version was agreed upon.

## Results

**Fig. 2:**
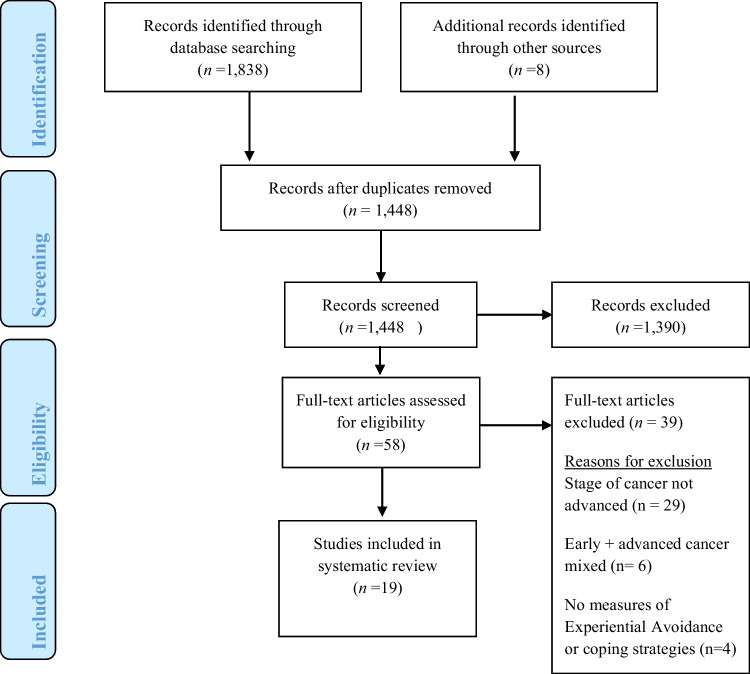
Flow diagram of literature search

From the 1448 retrieved reports, 19 met the inclusion criteria and were included in the review (Fig. 2). Thirteen reported quantitative data and 6 reported qualitative data. Before presenting these results in two respective sections, we detail how EA is variously described in the advanced cancer literature.

### EA Descriptors

**Fig. 3:**
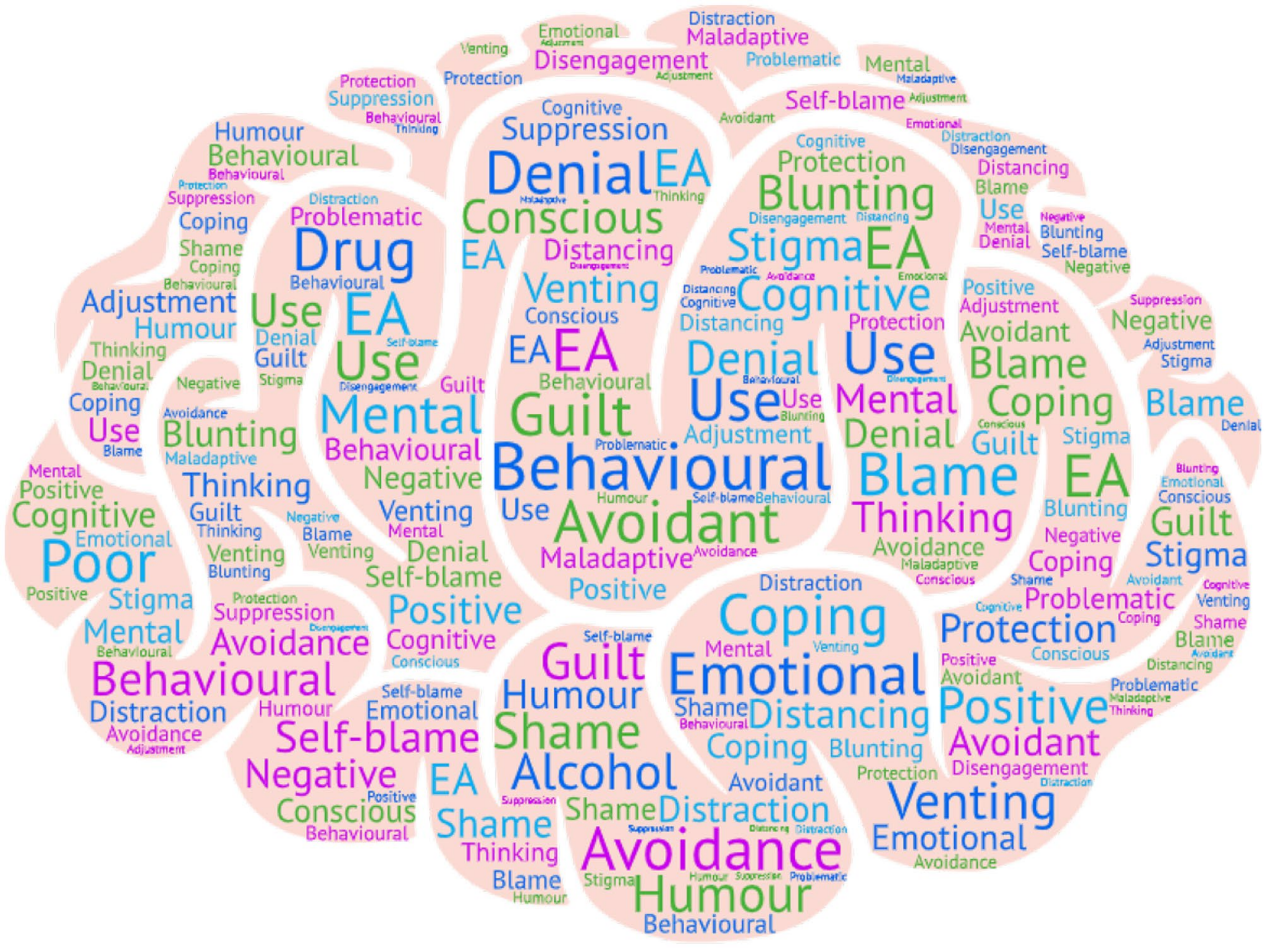
Word cloud of EA descriptors

A range of different terms were used to describe EA. A word cloud of EA descriptors depicts the most common words (larger font) and different terms used (Fig. 3). Emotional avoidance was often used [39, 43–45], and frequently in combination with behavioural avoidance [45–48]. Cognitive avoidance was rarely used alone [49] but usually with behavioural avoidance [50, 51] and emotional avoidance [52]. ‘Avoidant coping’ was also used as a descriptor for types of EA, but studies used different tools to assess this [45, 46, 50], making it difficult to determine what it encompassed within the terms of an individual study. For example, in Kershaw et al. [45], 6 different types of behaviour were classified as ‘avoidant coping’ using the Brief COPE [38] which included self-distraction, venting, humour, denial, behavioural disengagement, and alcohol/drug use. Once identified, these behaviours were not referred to again individually, but only collectively as ‘avoidant coping’. Alternative terms have also been used to describe similar constructs, for example, cognitive avoidance [49] and mental disengagement [51], demonstrating that the nature of the type of avoidance is not always clearly described in studies.

Different expressions of emotion were also described as ‘avoidant coping’. These included ‘venting’ [45, 46, 53] and ‘self-blame’ [44] which were found to be detrimental and associated with a negative appraisal of illness. Venting implies the process of dealing with emotions is problematic, and maladaptive [54]. It is less clear whether self-blame is a type of EA. In Nipp et al. [44], 38% of ex-smokers with lung cancer used self-blame whilst 28% used denial. Self-blame is linked with stigma and poor adjustment [55] and is also associated with shame and guilt. Guilt may render someone powerless and unable to galvanise themselves to actively cope with their disease, which may explain why it is included as a type of EA.

The descriptors found in the qualitative results included suppression of thoughts to protect themselves [56]. Distraction—to keep minds busy and provide relief from thoughts [2]. Distancing—to keep away from friends and family to avoid talking about cancer [2, 56], or only talking about normal things [57]. Blunting—helped to avoid negative information [58] or too much information [2]. Positive denial—helped people to feel positive and think optimistically [58] and to avoid negative thinking [57]. Conscious denial helped people refuse to think or acknowledge cancer [56, 59].

### Quantitative Results

#### Study Characteristics

Thirteen studies had a total sample of 1568 patients with advanced cancer, with a median sample size of 120 (range 32–350). Fifty-three percent of the patients were male, and mean ages across the sample ranged from 34 to 70 years. The participants were recruited from single hospital sites, outpatient clinics, and an inpatient palliative care unit [43, 44, 48, 51–53] and from multiple hospital sites, cancer centres, and satellite clinics in the same area [39, 45–47, 49, 50]. One study did not provide details about the identification of participants but gave details of recruitment by post [60] (Table 2).

**Table 2:** Characteristics and findings of studies on experiential avoidance in advanced cancer

**Authors, (year),** **country**	**No. in study advanced cancer/total sample**	**Site of participant recruitment**	**Patient group, demographic details**	**Study design; data collection methods; sampling**	**EA measure**	**Outcome measure**	**Study aims**	**Key findings**
Brown et al. [48] Australia	110	1 hospital in Sydney (Part of a larger study on psychosocial outcomes every 3 months for 2 years)	1. 55 years 2. 68% male 3. 100% melanoma (stage IV) 4. Not reported but reference to effects of treatment	Quantitative longitudinal; purposive	General Coping Strategies Scale COPE	PAC LASA QOL scales	Does emotion-focused coping increase distress as death approaches?	Psychological deterioration over time Avoidant coping did not change Did not support L + F’s theory
Costanzo et al. [52] USA	32/64	Hospital clinics in IOWA (does not say how many)	1. 62 years 2. 100% female 3. Ovary 72% Endometrium 14% Cervix 10% Fallopian tube 3% 4. 100% on chemo	Quantitative longitudinal (sample taken from a longitudinal study) analysis cross-sectional	COPE (28/60 items) IES	FACT-G POMS	Do people use more coping strategies? Is avoidant coping associated with distress and poorer QOL?	Disengagement + cognitive avoidance, strongly associated with poorer well-being and greater distressed mood
Couper et al. [49] Australia	156/367	7 public hospitals and practices in Melbourne	1. 70 (42−90) 2. 100% male 3. 100% prostate 4. Not reported	Quantitative descriptive; longitudinal baseline and 1 year; prospective	Mini MAC (20/29 items)	SF-36 BSI-53	To assess psychological distress at diagnosis and 1 year to see what psychosocial factors predict distress	Increased depression + anxiety at 1 year predicted by earlier anxiety, mental health, cognitive avoidance
De Faye et al. [53] Canada	52/52	Hospital, inpatient palliative care unit, day hospice	1. 66 (37−87) 2. 63% female 3. Breast 19.2% Lung 15.4% Bowel 15.4% Genitourinary 13.5% Gastro I 11.5% Head + neck 9.6% 4. Not reported	Difficult to understand sample as using interview methods but quantitative	Cheng et al. coping behaviours	BDI-PC	Determine patterns of coping across dimensions of distress (social, physical, existential)	Range of coping strategies to deal with stressors but not necessarily related to psychological distress
Kershaw et al. [45] USA	189 carer-patient dyads	4 large oncology centres and 3 satellite clinics	1. 54 (22−86) 2. 100% female 3. 100% breast cancer 4. Chemo 73% Radiation 24% Hormone 19.9% Bone marrow T 11.8%	(Part of an RCT on a family intervention) baseline measures prior to randomisation, cross-sectional	Brief COPE (12/14 items)	SF-36	Determine how women cope with advanced cancer find which strategies are assoc with QOL	Avoidant coping (religion, positive reframing, self-distraction, venting, and humour used more often than active coping Associated with lower mental QOL
Lutgendorf et al. [51] USA	33/95	University of Iowa Hospitals and Clinics (numbers not reported)	1. 63 (36−81) 2. 100% female 3. Ovarian 4. Surgery radiotherapy chemo	(Part of a 3-year longitudinal study) measures taken again at 1 year, longitudinal, prospective	COPE (36/60 items)	POMS FACT-G	Determine relationships between coping QOL and mood over 1 year following diagnosis	Disengaged coping associated with poorer doctor-patient relationships at 1 year; continued disengagement associated with poorer QOL and greater distress
Manne et al. [50] USA	98/189	2 large cancer centres	1. 57 (29−77) 2. 55% male 3. Breast 34% Colon/rectal 24% Prostate 20% Lung 9% 4. 75% Chemo 25% radiation	Quantitative description, longitudinal, convenience	Impact of Events Scale, IES	MHI (MH subscale used)	To test Creamer’s theory—avoidance is maladaptive way of dealing with trauma Determine assoc b/w intrusions, avoidance, disease stage + distress	Avoidance mediated b/w intrusive thoughts and psychological distress
Nipp et al. [44] USA	350	Hospital cancer centre	1. 64.9 2. 54% male 3. Lung 54% Gastrointestinal 45% 4. Chemo 79% Radiation 19%	(Part of an RCT on p/c) baseline measures taken prior to randomisation, cross-sectional	Brief COPE (7/14 items)	FACT- G HADS	Determine how people cope with incurable cancer Is there an association with mood and QOL?	Negative correlation between denial QOL + mood
Sherman et al. [46] USA	120/120	Tertiary treatment centre	1. 60 2. 72% male 3. Larynx 25% Lip + mouth 14.2% 4. 30 on treatment 30 within 6 mths	Quantitative description, cross-sectional survey, prospective	COPE (39/60 items) + Coping effort Breadth of flexibility IES	POMS	To determine coping at different phases of treatment and see if there is a relationship with distress	Different coping based on phase of tx Denial (*p* < .05), behavioural disengagement + withdrawing assoc with distress
Sumpio et al. [47] USA	121/121	Outpatients in a cancer hospital	1. (31−85) 2. 50% male 3. Colon 39% Pancreatobiliary 30% Lung 25% 4. 100% on chemo tx	Quantitative description: cross-sectional survey, convenience	Brief COPE	POMS–SF	To determine if coping and mood disturbance are affected by optimism, self-efficacy, symptom distress, and treatment complexity	Avoidant coping associated with disturbed mood, but not in the multivariate model
Trevino et al. [39] USA	53/53	Cancer institute	1. 33.89 (20−40) 2. 66% female 3. Breast 39.6% Lung, bone, pancreas, stomach—not reported Brain 13.2% 4. Not reported	Quantitative description: cross-sectional survey, convenience	Brief COPE	PG-12 MQOL	Determine coping strategies used by young adults and see if there is an association with distress	Proactive coping and distancing 1/3 of the variance Negative expression was positively associated with severity of grief
Van-Laarhoven et al. [60] Netherlands	59/151	No information about how participants were recruited or from where Questionnaire sent by post—only completed by 52% of p/c group	1 62 (no range) 2. 71 M 80 W 3 Breast 34% Prostate 16% Testes 11% Lung 9% Melanoma 6% Bowel 4% 4. Not on treatment	Quantitative description: cross-sectional survey	COPE easy (abbreviated Dutch version)	BDI- PC EORTC	Determine whether coping is associated with depression, hopelessness, and QOL	AV coping (denial, giving up) not beneficial re QOL Positively associated with depression + hopelessness

Key: 1) mean age, years (range); 2) male %; 3) cancer type; 4) treatment; 5) General Coping Strategies Scale, COPE (Weisman and Worden [103])

*PAC* Psychological Adjustment to Cancer Scale (Dunn et al. [76]), *LASA* *QOL* Linear Analogue Self-assessment Scales on Quality of Life (Priestman and Baum [104] and Hürny et al. [105]), *COPE* Coping Orientations to Problems Experienced (Carver et al. [27]), *IES* Impact of Events Scale (Horowitz et al. [24]), *FACT-G* Functional Assessment of Cancer Therapy-General (Cella et al. [40]), *POMS* Profile of Mood States (McNair et al. [64]), *Mini-MAC* Mini-Mental Adjustment to Cancer (Watson et al. [106]), *SF 36* Medical Outcomes Study Short Form 36 (Ware and Sherborne [107]), *BSI-53* Brief Symptom Inventory (Derogatis and Melisaratos [108]), *COPE* easy Dutch version of the COPE, *BDI-PC* Beck Depression Inventory for Primary Care (Beck et al. [109]), *Brief COPE* shorter version of the COPE (Carver et al. [38]), *MHI* Mental Health Inventory (Veit and Ware [110]), *HADS* Hospital Anxiety and Depression Scale (Zigmond and Snaith [65]), *PG-12* Prolonged Grief Disorder Scale (Prigerson et al. [75]), *MQOL* McGill Quality of Life Questionnaire (Cohen et al. [111]), *EORTC-QLQ C30* European Organisation for Research and Treatment of Cancer Quality of Life questionnaire (Aaronson et al. [112]), *DCI* Denial of Cancer Interview (Vos et al. [71])

Six studies used a comparative design, whereby coping was compared between early- and late-stage cancer patients [49–52, 60], and in 1, a comparison was made between patients and carers [45]. The participants were still in receipt of cancer treatments (chemotherapy, radiotherapy, hormone therapy, and surgery) in 10 studies [39, 43–47, 49–52]. No reporting of treatment occurred in 2 studies [48, 53], and in 1, the participants received no treatment [60] (Table 2).

Six studies were longitudinal designs [43, 48–52] of which 1 focused specifically on denial [43] and another on cognitive avoidance [49]. The aim of most studies was not to specifically examine EA, but to look at the different coping strategies people used to ascertain if there were any associations with mood, quality of life, and psychological distress. Six of the cross-sectional studies [39, 44, 46, 47, 60] were observational, whereby patients completed self-report measures of coping, psychological distress, and quality of life. A cross-sectional study [53] adapted and modified coping strategies using a structured interview method [61] based on hypothetical situations to develop a theoretical framework [62]. A form of coping called blunting was identified related to EA whereby threatening information was avoided or treated less seriously (Table 2).

#### Measures of Coping and Psychological Distress

The most frequently used outcome measures were the Coping Orientation to Problems Experienced (COPE; [27, 46, 52]) and the Brief COPE [38] which was used in 4 studies [39, 44, 45, 47]. The COPE is designed to measure ways in which people respond to stress and contains subscales of EA. The Brief COPE has been validated in incurable cancer and shown to have good psychometric properties [63]. Another measure that was used is the Impact of Events Scale (IES) [24] which is a measure of stress-related symptoms of avoidance and intrusion, and the IES was used in 3 studies [46, 50, 52]. Definitions of EA are taken from subscales of outcome measures with examples to provide clarity (Table 1).

Measures of psychological distress included the Profile of Mood States (POMS) [64], the Hospital Anxiety and Depression Scale (HADS) [65], the Brief Symptom Inventory (BSI) [66], and the Beck Depression Inventory (BDI) [67], as well as various subscales of quality-of-life measures like the Short Form Survey (SF-12) [68] that assess mental well-being.

### Quality Assessment of Quantitative Studies

We present our quality assessment of included quantitative studies using the QualSyst tool [41] in Table 3. Overall, the quality of the studies was good with some improvements needed in specific areas. Of the 13 quantitative studies, 6 used longitudinal survey methods [43, 48–52] and 4 of these had a comparison group [49–52]. Six studies used cross-sectional survey methods [39, 44–47, 60], and 2 of these had a comparison group [45, 60]. One study [53] used an interview method to gather data.

**Table 3:**
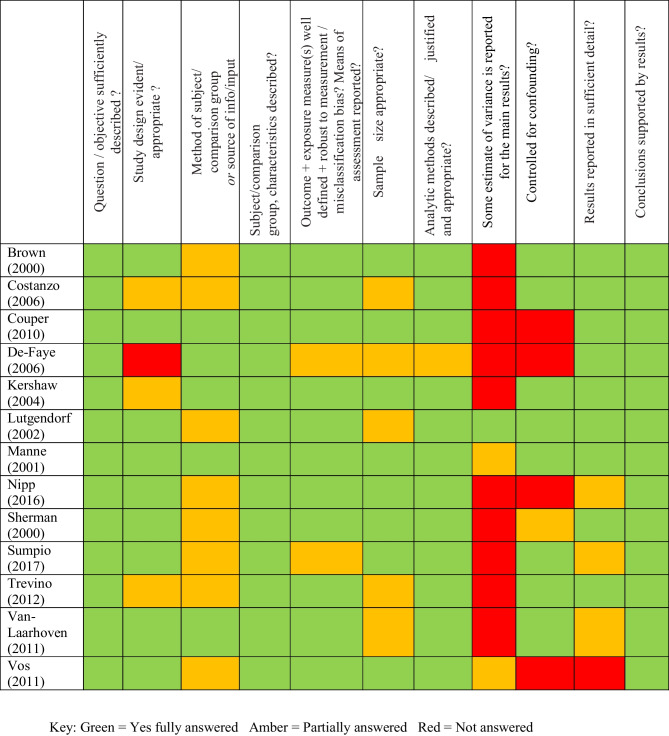
Quality assessment checklist of quantitative studies

All 13 studies had clear overall objectives, which were achieved; analytic methods were justified and appropriate; and the conclusions of the studies were supported by the results. Study design was generally good with 9 studies having an appropriate design for their research. Three studies had some limitations [44, 45, 52], and in one study [53], the design was unclear.

Sample sizes of advanced cancer patients ranged from 32 to 350. Five of the studies [39, 51–53, 60] had relatively small sample sizes ranging from 32 to 59 participants.

Eleven studies provided full information about the measurement of outcomes with only 2 studies providing partial information [47, 53]. Results were reported in sufficient detail in 9 studies, 3 provided partial information [44, 47, 60], and only 1 did not supply enough information [43]. In almost all studies, analytic methods were well described, justified, and appropriate. In just 1 [53], however, it was unclear how interview data concerning coping behaviours was incorporated into a theoretical model of coping behaviour.

The main critique of the studies (*n* = 10) was the complete lack of variance reported. Partial information was provided in 3 studies [39, 43, 50] with only 1 providing full information [51]. The strength of the results therefore could not be properly assessed or statistically estimated due to chance [69]. Eight studies provided partial information about comparison variables and 5 provided full information [45, 49, 50, 53, 60]. No controls for confounding were found in 4 studies [43, 44, 49, 53], and limited information was provided in 1 other [46], which could have affected the validity of the results.

### Types of Avoidance and Associations with Psychological Distress

#### Cognitive Avoidance

Four longitudinal studies assessed whether coping strategies were associated with psychological distress and quality of life [49–52]. Prostate cancer patients assessed as anxious at baseline who engaged in cognitive avoidance remained anxious at 1 year [49]. Distress was significantly predicted by anxiety, cognitive avoidance, and lower anxious pre-occupation [49]. A strong correlation was found at baseline between intrusive thoughts and distress. Behavioural attempts to avoid thoughts at 3 months related to psychological distress at 6 months, so avoidance acted as a mediator. Attrition at 6-month follow-up was high, which may represent a selection bias.

Two longitudinal studies compared women with early and advanced gynaecological cancers who had received extensive treatment [51, 52]. In Lutendorf et al. [51], although sample sizes were small (*n* ≤ 33), cognitive avoidance (mental disengagement) at baseline was significantly associated with a poor relationship with the doctor at 1 year and behavioural disengagement was associated with greater distress. Similarly, giving up attempts to cope were common and strong associations were found between cognitive, behavioural, and emotional avoidance strategies with greater anxiety and depressed mood [52]. However, despite data from this study being longitudinal, the analysis was cross-sectional.

#### Behavioural Avoidance

De-Faye et al. [53] found behavioural avoidance was used to cope with the social domain of stress. Distraction was used 53% of the time and diverting attention 58% of the time. No associations were found with psychological distress, but interview methods to categorise coping behaviours were unclear which may have reduced associations.

Three cross-sectional studies analysed the effects of coping strategies on mood [45, 47, 60] using the Brief COPE [38], in which avoidant coping was defined as self-distraction, denial, behavioural disengagement, self-blame, and venting. In Sumpio et al. [47], avoidant coping was associated with greater symptom distress, negative appraisal of illness, and greater mood disturbance. Van-Laarhoven et al. [60] compared curative and palliative patients and found behavioural disengagement and denial were positively associated with depression and hopelessness in the palliative group. Expression of negative emotions through venting had a negative predictive effect on emotional functioning in the group.

Kershaw et al. [45] found advanced breast cancer patients used both active and avoidant coping strategies. Small to medium correlations between coping strategies and physical and mental quality-of-life variables showed behavioural disengagement, denial, substances, and venting were significantly associated with more symptom distress and lower mental quality of life. Participants in this study were part of a large interventional randomised controlled trial, so they may not have been typically representative of this population.

Four different stages of treatment were examined in a cross-sectional study of coping in people diagnosed with head and neck cancer [46]. Patients engaged in behavioural disengagement, denial, and emotional ventilation frequently when undergoing treatment and up to < 6 months afterwards, which were associated with psychological distress.

#### Emotional Avoidance

Vos et al. [43] explored whether denial had an effect on mood and quality of life over time in lung cancer. The Denial of Cancer Interview (DCI) assessed levels of denial [70]. Moderate deniers compared to low deniers had significantly less anxiety and depression. Increasing deniers had more distress initially, which decreased later. In addition, moderate and increasing deniers had a better quality of life than low deniers.

Nipp et al. [44] used a cross-sectional study to evaluate the relationship between coping, mood, and quality of life in a large group (*n* = 350) of newly diagnosed participants with incurable lung and gastrointestinal cancers. Higher denial and self-blame (*B* = 0.580, SE = 0.1666, *P* < 0.001) were significantly correlated with higher depression and anxiety scores.

Although both Nipp [44] and Vos [43] employed samples of people newly diagnosed with advanced cancer, there are crucial differences in the definition and measurement of denial as well as the method used that limit comparison but may in part explain the differences in findings. For example, Vos [43] used a comprehensive 11-item scale the Denial of Cancer Interview (DCI) [71] based on Weisman and Hackett’s definition [72, 73]. Nine items were self-report and 2 were assessed by a clinician at 4 time points 8 weeks apart to provide longitudinal data. Nipp [44] only used a 2-item self-report subscale of the Brief COPE [38] to measure denial cross sectionally based on Lazarus and Folkman’s model of coping [1] and Carver and Scheier’s model of self-regulation [74].

Trevino et al. [39] investigated how a predominantly female (66%) sample of young adults [20–40 years] with heterogeneous cancers coped with a poor prognosis. ‘A factor analysis of subscales of the Brief COPE identified 6 coping factors of which Negative Expression was one. Denial, venting, and self-blame loaded onto the Negative Expression factor. After controlling for depression, anxiety, and other confounders coping by negative expression was directly related to grief and losses to cancer identified on the Prolonged Grief Disorder Scale (PG-12; [75].

Melanoma patients’ psychological adjustment, coping, and quality of life were examined over 2 years by Brown et al. [48]. Avoidant coping (distraction, eating, substances) had a significant negative effect on mood. In another study psychological adjustment to cancer as measured using the Psychological Adjustment to Cancer scale (PAC) [76] found people who isolated themselves and tried not to let people know about their cancer experienced significantly lower mood.

### Qualitative Results

#### Study Characteristics

Six qualitative studies were identified. Of the total number of participants (*n* = 178), 136 had advanced cancer of which 109 were female. Characteristics of the included studies are summarised in Table 4. Different approaches to data analysis included phenomenology/interpretive phenomenological approach [56, 57], grounded theory [2, 58, 77], and qualitative descriptive thematic analysis [59].

**Table 4:** Characteristics and findings of studies on experiential avoidance in advanced cancer

**Authors (year),** **country**	**Proportion of participants with advanced cancer/total sample**		**Site of participant recruitment**	**Patient group, demographics**	**Study design: data collection methods; sampling**	**Main aims of study**	**Key findings in relation to experiential avoidance**
Lobb et al. [2] Australia	27/27		Medical radiology/oncology and palliative care services at 3 urban hospitals	1) 63 (21−88) 2) 59% female 3) Bowel 38% Breast 23% Lung 19% Prostate 8% Other 12% 4) Not reported	Qualitative grounded theory, semi-structured interviews, convenience	How patients with advanced cancer cope with uncertainty	Distraction to keep busy and avoid thoughts Distraction with other things to avoid talking about illness to family + friends Minimised illness by comparing themselves favourably with others Focused on positive information, avoided negative information about prognosis
Luoma et al. [56] Finland	25/25		Large multicentre chemotherapy centre NB part of a clinical trial of chemotherapy	1) Not reported 2) 100% female 3) Breast 100% 4) 100% receiving chemotherapy	Qualitative phenomenology, semi-structured interviews, purposive	How patients with advanced breast cancer describe the meaning of QOL	Used distraction, TV, to avoid thinking about cancer Avoided thinking about cancer to protect themselves Maintained relationships with friends by not talking about cancer
Lam et al. [77] Hong Kong	16/42 (NB drawn from a quantitative longitudinal study on distress)		Public clinical oncology/surgical units (no details of n^o^s)	1) 49 (30−73) 2) 100% female 3) 100% breast 4) No active treatment	Grounded theory, semi-structured interviews, purposive	Living with advanced breast cancer how meanings compare between those with low/stable distress with those with persistent distress	Acceptance helped patients not to ruminate Those with persistent distress and minimal support avoided talking to friends or neighbours about cancer and tried to hide it
Kvale [57] Norway		16/20	In-patients on a cancer ward of a hospital	1) Not reported (40−70) 2) 50% female 3) Different cancers but no details 4) Receiving curative or palliative treatment	Qualitative phenomenology, open-ended questions, purposive	Do inpatients on a cancer ward want to talk to nurses about their feelings and emotions about the disease and the future?	Distancing to avoid thinking about illness Do not always want to talk to nurses or family about feelings or illness, but ordinary things Want to be considered normal people with interests; their identity is not their cancer Support from families helpful
Power et al. [58] Canada		18/30	Gynaecological oncology clinic in a cancer hospital	1) 59 (37−79) 2) 100% female 3) Epithelial ovarian cancer 4) 50% in treatment	Qualitative grounded theory, semi-structured interviews in person/phone, convenience	To identify types of coping; psychological distress; support; at different stages and phases of ovarian cancer	Avoidance + blunting of information to prevent people from dealing with information Humour helped people to be optimistic Positive denial used when information was negative
Liao et al. [59] Taiwan		34/34	Outpatient departments in a hospital and medical centre (selection by researcher)	1) 58 (35–82) 2) 100% female 3) Lung cancer 4) 82% in treatment	Qualitative description, open-ended questions with prompts, purposive	To find out which coping strategies lung cancer patients used to manage psychological distress	Avoiding thinking about the cancer provided temporary relief Avoided talking about cancer by concealing it or avoiding social contact Positive thoughts used to counter negative ones

Key: 1) mean age, years (range), 2) female %, 3) type of cancer, 4) receiving treatment

**Table 5:**
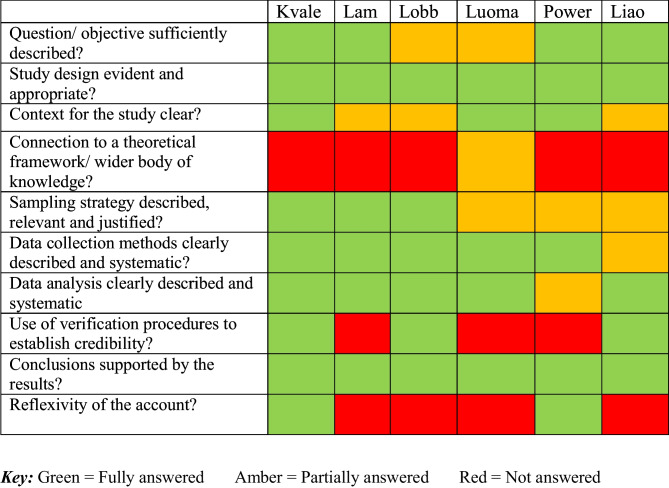
Quality assessment checklist of qualitative studies B

#### Quality Assessment of Qualitative Studies

We present our quality assessment of included studies using the QualSyst tool [41] in Table 5. The quality of studies varied in relation to the amount of detail given of their sampling strategies. Four used purposive sampling [56, 57, 59, 77] and 2 adopted a consecutive approach [2, 58]. In 1 study, the researcher selected patients to participate [59], which could have introduced bias. Lam et al. [77] used a retrospective design that meant participants could have been subject to recall bias. Two studies in advanced breast cancer used participants already participating in longitudinal trials: 1 on effects of chemotherapy [56] and 1 on psychological distress following diagnosis [77].

Studies also lacked detail of time since diagnosis [2, 56, 57], and some patients may have been living with their cancer for longer and may have developed more adaptive coping strategies that included actively dealing with a problem by seeking information, enlisting instrumental support, problem solving and forward planning, positively reinterpreting difficulties, and willingly accepting experiences. The objectives of the studies and their design were well described, but scant detail was provided of the context and setting of the studies [2, 59, 77]. In addition, the majority of studies lacked a theoretical framework or disciplinary body of knowledge to inform their methods and research processes [2, 57–59, 77].

Most studies did not detail how findings may have been influenced by researcher bias apart from one in which the researcher selected patients [77]. Reflexivity of the researcher was only considered in two studies [57, 58] with consideration of how their researcher characteristics or methods could impact upon the research. Generally, a sufficient description of data analysis methods was provided, and the conclusions reached were supported by the results.

### Thematic Synthesis

The aims of our thematic synthesis were first to explore participants’ coping strategies and identify EA and second to explore participants’ rationale for engaging in EA. EA was used to help mitigate the effects of cancer, and five themes were identified: [1] protection from distress, [2] preservation of identity, [3] maintenance of normality, [4] control of information, and [5] maintenance of optimism and hope.

#### Protection from Distress

People were anxious not to think or talk about their cancer [2, 56, 58, 59, 77] so used different cognitive and behavioural avoidance techniques to shield themselves from emotional distress. Suppression of thoughts was one technique to protect against experiencing uncomfortable or difficult thoughts.

*I try not to think about it. It’s a way to protect myself that I refuse to think about it *[56].

Distraction helped occupy peoples’ minds and provided temporary relief from negative thoughts, until they resurfaced:

*I might just get in the car and do my shopping and it’s gone (thoughts) ….and I just put it away and I don’t want to think about it anymore until somethings else comes up *[2].

Distancing helped people avoid social situations where they may be reminded of their illness. Patients with incurable lung cancer [59] who felt overwhelmed by their illness described distancing as a means of avoiding distress.

*I’m afraid that I’ll go to pieces if I talk about my cancer with others *[59].

By purposefully avoiding social contact, individuals side-stepped conversations that may require them to confront and express difficult emotions about their cancer.

Even though one participant recognised that avoiding her friends distressed her, she continued to do this:

*I refuse to see any of my old friends, because I don’t want them to gossip about me and I don’t want to repeatedly answer their questions about cancer. This makes me sad *[59].

#### Preservation of Identity

Participants with breast cancer [56] avoided talk about their cancer, as they suspected people would behave differently towards them if they knew of their diagnosis. One participant highlighted the reasons why:

*I don’t want to tell anyone I’m like this. I don’t want to talk about it to strangers, cause I feel that people would start to feel sorry for me…..I don’t want any of that. I don’t think that I’ve changed that much as a person. I’m still the same person, even if I’ve got this disease. That’s probably why I don’t want to talk about it to anyone *[56]*.*

People did not wish their identity to be tainted by their cancer diagnosis, and they did not want to receive sympathy from strangers. Perceptions people with advanced cancer held about their illness challenged the way they thought about themselves and avoiding talking about it to strangers enabled some of the participants to preserve their identity so that their illness did not define them. Avoiding talking to people they knew maintained these relationships on their old terms, based on the person’s pre-cancer identity.

#### Maintaining Normality

One study investigated whether hospitalised advanced cancer patients wanted to talk about difficult emotions with their nurses [57]. Participants revealed that they preferred sometimes to talk about mundane things, or things that were part of everyday ‘normal’ life, rather than their illness:

*I want to live as normally as possible. I want to talk about what I am doing at home. They (nurses) know much about me. I want to talk about ordinary things *[57].

Crucially, the patient wants to be known as a person, valued as such, and with a life outside the hospital, not just as a cancer patient with specific needs.

Similar findings were reported in a study of how mixed cancer patients manage uncertainty. A participant was keen to circumscribe talk about her cancer so that it did not pervade her life:

*I try to avoid talking about it as much as possible because you’ve got to focus on other things. And as much as friends and people say “how are you?” (I say) “I’m alright” …. you’re going, no, I don’t want to talk about this again *[2].

Avoidance of talking about the disease not only helped the participant avoid potentially upsetting reminders but also helped to maintain and portray a semblance of normality with others.

#### Control of Information

Participants who were fearful of receiving negative information about their illness and prognosis sought to control information in various ways [2, 58]. A technique called ‘blunting’ [58] was used by women with gynaecological cancers to avoid exposure to negative information:

*I have been non-interested in hearing about ovarian cancer stuff because I know how negative it is. So, I have not paid attention to it. I don’t particularly want to know anyway *[58].

A participant who refused to listen to negative information likened the process to denial:

*I think maybe it is part of the denial process in many ways; that you know you have got something really serious and you don’t really want to know how serious it is in some ways *[58]*.*

In a study of individuals with heterogenous cancers [2], participants were worried that too much information would make them feel worse, so tried to limit the amount of information they received:

*I just want to know what I need to know, because I think for me too much information is not going to help me, it’s just going to make me more stressed *[2].

and control the tenor of this:

*I need to hear something that’s a bit confident, you know that gives me confidence, you know to keep going *[2].

#### Maintenance of Optimism and Hope

Maintaining an optimistic outlook and not worrying about the future, despite being aware of their diagnosis, helped some people cope with the risks and realities of their illness [56–59].

Some engaged in positive denial whereby they counted on a return to their former health state before their cancer.

*I feel very optimistic and very confident at this stage. I feel extremely positive myself and I think that makes a big difference as to how you handle things *[58].

*I don’t take the cancer seriously. I believe that I can be cured. I have not been thinking negative thoughts about illness. I try to be positive then it is much easier for me *[57].

Maintenance of optimism and hope was nuanced across the studies as people adopted different strategies. Conscious denial [56, 59] occurred when people actively refused to think about or acknowledge their cancer, to reassure themselves they were going to be alright:

*I try not to think about it. It’s a way to protect myself that I refuse to think about it. …If I start thinking about this illness, I’m lost and I haven’t got anything left in life than this awful disease *[56].

Another participant draws on their imagination to deny the reality of their cancer and perhaps derives some relief from this:

*I just try to not think about my illness. I tell myself I am okay, and my disease is gone *[59].

## Discussion

This systematic review of quantitative and qualitative research examined the nomenclature of EA in advanced cancer and the processes that might indicate EA, and explored associations between EA and psychological distress and reasons for engagement in EA. EA is a broad term that covers a variety of coping strategies in the psycho-oncological literature [19, 20]. We have defined and classified the terminology used to describe EA; this covers cognitive, behavioural, and emotional avoidance, and includes the term ‘avoidant coping’ [54]. Although a range of terms are used for EA, no specific scales are used to measure it. EA in advanced cancer patients may be associated with an exacerbation of psychological distress and behaviours which perpetuate emotional problems, thus preventing psychological adjustment, although in a small number of people, it may be helpful in the short term.

### Association Between EA and Psychological Distress

There is evidence of an association between EA and psychological distress. However, as no studies in advanced cancer have specifically examined EA as a primary outcome, definitive conclusions need to be treated with caution. There are no standardised methods to study EA in advanced cancer. Trials to date cover a range of cancers, use a range of methodologies, and lack standardised outcomes. Furthermore, despite the research being rated of good quality due to a low risk of bias, limitations included a lack of detailed reporting of information, a lack of variance and controls for confounders, and research processes that were not always based on a theoretical framework or a body of knowledge. Measures to assess coping were also often not complete [44–46, 49, 51, 52, 60]. Although existing measures have been adapted for assessing EA, only the Brief COPE has had its psychometric properties evaluated in incurable cancer [63]. Livneh [22] suggests that despite an association between avoidant coping and poorer psychosocial adaptation in chronic illness (including cancer), studies need to be strengthened with respect to reporting illness severity and sociodemographic details, and using psychometrically tested scales [22].

The relationship between the cancer type, stage, and EA and its impact on mood is uncertain [39, 44–47, 60]. In two studies, it was suggested that EA is the mediator between anxiety and distress [49, 50]. By contrast, EA did not appear detrimental as a short-term strategy [50]. Differences in EA were found which may be accounted for by tumour groups. Higher levels of distress are present in breast, gynaecological, prostate, and head and neck cancers [78]. High levels of denial (emotional avoidance) [43, 44] are associated with increased anxiety and depression, and symptoms of distress [45, 47] and grief [39], as well as a poorer quality of life [44]. By contrast, Vos et al. [43] found that as denial increased distress decreased [43].

A more recent study found that older patients used more denial than younger patients and denial was weakly correlated with problem-based coping [79]. These coping strategies appear contradictory and imply people may fluctute in their choice of coping strategy or combine contrasting strategies. Overall, denial appears to be associated with increased distress, but it is difficult to be sure of the direction of this relationship.

### Reasons Why People with Advanced Cancer Engage in EA

Numerous factors affect the way people cope with advanced cancer: cancer type; extent of disease; physical symptoms and function; emotional well-being; available resources; and environment [80]. Progression of disease and its diagnosis and treatment can cause repetitive and cumulative trauma leading to anxiety, depression, and post-traumatic stress disorder [81, 82]. EA may be used to reduce or regulate distress experienced in cancer particularly when a person feels overwhelmed and not able to cope [83]. EA can be adaptive if used in the short term to give people some respite from distress and help them to gather resources [84]. Continued avoidance of difficult experiences rather than direct contact, however, is maladaptive, if used over time, as it requires a large allocation of resources [14] and impairs functioning [85]. Acceptance of experiences as they occur without defence is more adaptive as functioning is preserved.

A review found disengagement and avoidance helped people to minimise cancer; control the illness experience; express emotions; and create meaning [86]. Pooling of qualitative data from our review found similar results, as EA was primarily used to protect people from distress and having to confront or express difficult emotions [2, 56, 58, 59, 77]. Avoiding communicating about cancer as well as reminders of it helped protect people’s previous relationships and preserve identity [56], as well as maintain normality [2, 57]. A reluctance to face difficult emotions meant people tried to control the information they received to avoid exposure to negative information [2, 58]. Positive denial enabled people to only pay attention to positive information and ignore any negative information about the seriousness of cancer or extent of disease which helped them to maintain hope and optimism about the future [56–59]. This bears similarities to elements of adaptive coping in the oncology literature when people positively reframe their thoughts by looking for something good in what is happening to help them cope which can enhance mood and quality of life [87].

### Strengths and Limitations

This is the first systematic review to describe EA in advanced cancer and suggests an association between EA and psychological distress. Although the literature search was completed in October 2019 an updated search only revealed 1 more relevant paper [79]. Due to the complex nature of the topic, both quantitative and qualitative data were synthesised to provide an exhaustive account of EA in advanced cancer. Our use of broad search terms covering different types of cognitive, behavioural, and emotional avoidance has contributed to an elucidation of EA in the advanced cancer population. However, this review only included peer-reviewed published papers in English. Grey literature may have revealed additional findings on this topic.

A limitation of the work in this field is the heterogeneity of constructs and lack of a clear definition of EA in advanced cancer making the comparison of studies difficult.

### Clinical and Research Implications

In the clinical setting, interventions are needed to assist advanced cancer patients to be more willing to accept uncomfortable or difficult feelings. Conventional therapies such as cognitive behavioural therapy have struggled to address patients’ anxieties because they relate to numerous rational stressors experienced at this stage [88]. ACT states that some psychological pain is inevitable in cancer and forms part of the experience of being human which cannot be avoided, but the suffering that may result from avoiding experiences is optional and can be addressed [89, 90].

ACT [28] focuses on the relationship between behaviours and all three types of avoidance, (cognitive, emotional, and behavioural), and may be a particularly well-suited intervention in advanced cancer. Research on the implications of EA in the aetiology and maintenance of diverse forms of psychopathology has grown considerably over the last 10 years, yet the contribution of EA to cancer-related distress has received very little attention in the advanced cancer population. A reason for this could be due to a lack of a comprehensive and standardised definition of EA in cancer.

We therefore define EA as behaviours whose function is to reduce the person’s contact with unwanted internal experiences. The distortion of facts such as wishful thinking, denial, and blunting is incorporated within this definition as their function is to alter the internal experience the person is in contact with, but EA does not incorporate self-blame or venting as their function is not to avoid unwanted internal experiences.

Greater clinical awareness of the complexity of EA behaviours is needed, and clinicians and researchers need to define EA precisely and be aware of its function in the short and long term.

The Acceptance and Action Questionnaire (AAQ) [30] and the (AAQ-II) [91] have traditionally been used to assess EA amongst cancer survivors [34, 92–96], but these have not been validated in advanced cancer. The AAQ-II [91] is limited, because it does not solely measure EA but measures psychological inflexibility instead which includes acceptance as well as EA [91, 97, 98]. The Multi-Dimensional Experiential Avoidance Questionnaire (MEAQ) [99] has been developed as a more comprehensive measure of EA, designed to assess six different elements of EA [100, 101]. However, future research needs to test and validate this measure in an advanced cancer population.

Furthermore, future qualitative research focused on exploring EA can build on these findings and explore the nuances of how people engage in EA and the perceived impact of this.

## Conclusion

People with advanced cancer engage in EA which may increase psychological distress if used rigidly and repeatedly [102]. Qualitative data suggests EA may be beneficial in the short term to those who feel overwhelmed, as it helps people continue to function through control of information, maintenance of normality, and an optimistic outlook, but in the longer term, it can impair function and limit engagement in life. A greater awareness of EA perpetuating emotional problems and preventing psychological adjustment is needed amongst clinicians.

Future work into EA should define the advanced cancer diagnosis and the time from diagnosis, and assess the type of EA with a standardised measure to enable treatment to target elements of EA.

### Supplementary Information

Below is the link to the electronic supplementary material.

Supplementary file1 (DOCX 13 KB)

## Declarations

### Statement Regarding the Welfare of Animals, Studies with No Animal Subjects

This article does not contain any studies with animals performed by any of the authors.
