# Supplementary material for: Experiential Avoidance in Advanced Cancer: a Mixed-Methods Systematic Review
Source: Int J Behav Med. 2022 Oct 25;30(5):585–604. doi: 10.1007/s12529-022-10131-4 (PMC10522753; doi:10.1007/s12529-022-10131-4)
Supplement: Supplementary file 1 — Supplementary file1 (DOCX 13 KB) [file 12529_2022_10131_MOESM1_ESM.docx]

**Supplementary material 1. Search strategy for systematic review**

**Subject headings**

Carcino* adj3 (advanced or incurable or palliative or terminal or late stage or end stage)

Cancer* adj3 (advanced or incurable or palliative or terminal or late stage or end stage)

Malignan* adj3 (cancer* or carcino* or tumo?r)

Metasta* adj3 (cancer* or carcino* or tumo?r)

Neoplas* adj3 (cancer* or carcino*or tumo?r)

Denial (psychology) or fantasy/or repression, psychology

Adaptation, psychological/or emotional adjustment

Defence mechanisms/or “denial (psychology)”/ or fantasy/ or repression, psychology/

**Keywords**

“Avoidance coping”

Avoid*adj2 (coping)

Avoid* adj3 (thought* or emotion* or feeling* or experience*)

Avoid* adj2 (cognitive or behavio?ral)

Adapt* adj3 (psychol*)

Adjust* adj3 (psychol*)

Coping adj3 (maladaptive)

Coping* adj3 (strategy* or pattern* or style or behavio?r*)

Disengage* adj3 (cognitive or behavio?ral*)

Distancing adj3 (cognitive or behavio?ral*)

Escap* adj3 (thought* or emotion* or feeling* or experience*)

Emotion* adj3 (adjust*)

Experien* adj3 (avoid*)

Suppress* adj3 (thought* or emotion* or feeling* or experience*)

Unwanted adj3 (thought* or emotion* or feeling* or experience*)

Psychol* adj3 (flexibility)

Maladpative adj3 (coping)

Accept* adj3 (experience* or thought* or emotion* or feeling*)

Non accept* adj3 (experience* or thought* or emotion* or feeling*)
